# Inhibition of polycomb repressive complex 2 by targeting EED protects against cisplatin‐induced acute kidney injury

**DOI:** 10.1111/jcmm.17447

**Published:** 2022-06-23

**Authors:** Chao Yu, Tingting Li, Jialu Li, Binbin Cui, Na Liu, George Bayliss, Shougang Zhuang

**Affiliations:** ^1^ Department of Nephrology, Shanghai East Hospital Tongji University School of Medicine Shanghai China; ^2^ Department of Medicine, Rhode Island Hospital, and Alpert Medical School Brown University Providence Rhode Island USA

**Keywords:** acute kidney injury, apoptosis, cisplatin, EED, EED226, EZH2, p53, polycomb repressive complex 2

## Abstract

Polycomb repressive complex 2 (PRC2) is a multicomponent complex with methyltransferase activity that catalyzes trimethylation of histone H3 at lysine 27 (H3K27me3). Interaction of the epigenetic reader protein EED with EZH2, a catalytic unit of PRC, allosterically stimulates PRC2 activity. In this study, we investigated the role and underlying mechanism of the PRC2 in acute kidney injury (AKI) by using EED226, a highly selective PRC2 inhibitor, to target EED. Administration of EED226 improved renal function, attenuated renal pathological changes, and reduced renal tubular cell apoptosis in a murine model of cisplatin‐induced AKI. In cultured renal epithelial cells, treatment with either EED226 or EED siRNA also ameliorated cisplatin‐induced apoptosis. Mechanistically, EED226 treatment inhibited cisplatin‐induced phosphorylation of p53 and FOXO3a, two transcriptional factors contributing to apoptosis, and preserved expression of Sirtuin 3 and PGC1α, two proteins associated with mitochondrial protection in vivo and in vitro. EED226 was also effective in enhancing renal tubular cell proliferation, suppressing expression of multiple inflammatory cytokines, and reducing infiltration of macrophages to the injured kidney. These data suggest that inhibition of the PRC2 activity by targeting EED can protect against cisplatin‐induced AKI by promoting the survival and proliferation of renal tubular cells and inhibiting inflammatory response.

## 1 INTRODUCTION

Acute kidney injury (AKI) is a common critical clinical state in which renal function declines rapidly^1^ and in which mortality can be as high as 50% or more.^2^ AKI is caused by a variety of insults, including ischemia/reperfusion, infection or exposure to various nephrotoxins.^3^, ^4^ Among nephrotoxins, cisplatin is an effective chemotherapeutic drug for treating various solid tumors, but is associated with nephrotoxic effects in 30%–40% of patients to whom the drug is administered.^5^ Cisplatin‐induced AKI is a critical problem that limits cisplatin's clinical application. In general, there is so far no specific treatment for AKI. Identifying the factors and mechanism behind cisplatin‐induced AKI will be beneficial to the development of new treatments for AKI.

Previous studies have demonstrated that apoptosis of renal proximal tubular cells is the major mechanism of AKI induced by cisplatin.^6^, ^7^, ^8^, ^9^ Cisplatin‐induced apoptosis can occur through activation of both intrinsic pathways with endoplasmic reticulum and/or mitochondrial dysfunction and extrinsic pathways with the activation of death receptors, leading to transcriptional activation of p53, a well‐known tumor‐suppressor protein, and subsequent upregulation of several targets including Bax.^10^ Located in mitochondria, Bax can cause mitochondrial membrane damage, resulting in caspase‐dependent apoptosis.^11^, ^12^, ^13^ In contrast, deacetylation of p53 by Sirtuin 1 (Sirt1), a histone deacetylase, can reduce apoptosis.^14^ In line with this observation, administration of resveratrol, a Sirt1 activator, inhibits AKI and improves renal function^15^; increased expression of histone deacetylase SIRT3, another form of SIRT family that is specifically expressed in the mitochondria, can also protect against cisplatin‐induced kidney damage by improving the dynamics of cell mitochondria.^16^, ^17^, ^18^ These findings suggest that histone‐mediated epigenetic modifications are closely related to cisplatin ‐induced renal tubular cell apoptosis and AKI.

Epigenetic modification refers to gene changes caused by post‐translational modifications of gene products without changes in DNA coding, thereby regulating the transcription of target genes and cell function.^19^ During the process of AKI, several epigenetic modifications such as methylation, phosphorylation, ubiquitination occur in histone and non‐histone proteins.^3^ Histone methylation is induced by activation of multiple histone methyltransferases.^20^ Among them, our recent studies showed that EZH2, a H3K27 methyltransferase, contributes to renal tubular damage and AKI induced by multiple insults, including cisplatin.^21^, ^22^ EZH2 is a subunit of polycomb repressive complex 2 (PRC2), which is composed of four core subunits (EZH2, EED, SUZ12, RbAp46/48).^23^, ^24^, ^25^ In this complex, EZH2 lacks enzymatic function on its own, but can gain histone lysine methyltransferase activity that catalyzes methylation of histone H3 at lysine 27 (H3K27) when it complexes with other non‐catalytic subunits of the PRC2 complex, in particular EED. EED binds to H3K27me3 and EZH2, allosterically promoting PRC2 histone methyltransferase (HMT) activity. Thus, PRC2 catalyzes methylation of histone H3 at lysine 27 (H3K27) through EZH2, with EED functioning as an epigenetic “reader” that is essential for full catalytic activity of PRC2.^26^, ^27^, ^28^ Although our recent studies have shown that inhibition of EZH2 protects against AKI in murine models,^21^, ^22^ the overall role of the PRC2 and the biological functions of EED in AKI remain unclear.

In this study, we examined the effect of PRC2 inhibition on AKI and mechanisms involved by employing EED226, a highly selective PRC2 inhibitor that directly binds to the H3K27me3 binding pocket of EED, resulting in loss of PRC2 activity, in a murine model of cisplatin‐induced AKI. Our results demonstrated that EED226 can effectively reduce H3K27me3 levels, inhibit renal tubular cell apoptosis and alleviate AKI due to cisplatin. Additionally, treatment with EED226 or siRNA‐mediated silencing of EED was effective in reducing renal tubular cell apoptosis in cultured cells exposed to cisplatin. Collectively, these results demonstrate the feasibility of utilizing EED‐targeted inhibitor to interrogate PRC2 biology, and as a potential therapeutic treatment of AKI.

## 2 MATERIALS AND METHODS

### 2.1 Antibodies and reagents

Antibodies to EZH2 (^#^5246), H3K27me3 (^#^9733), EED (#85322), cleaved caspase3 (#9664), Caspase3 (^#^9662), p‐p53 (Ser 15) (^#^9284), p53 (^#^2524), p‐STAT3 (Tyr 705) (^#^9145), STAT3 (^#^9139), Bax (^#^2772), FoxO3a (^#^12829), p‐FoxO3a (Ser 253) (^#^9466), p‐NF‐κB (Ser 536) (^#^3033), NF‐κB (^#^8242), p‐MLKL (Ser345) (^#^37333), MLKL (^#^37705) and p‐RIPK3 (Thr231/Ser232) (^#^91702) were purchased from Cell Signaling Technology (Danvers, MA, USA). Antibodies to p21 (^#^sc‐6246), NGAL (^#^sc‐515,876) were obtained from Santa Cruz Biotechnology (Santa Cruz, CA, USA). PGC‐1α (^#^ab54481) antibody was purchased from Abcam, Inc.(Cambridge, MA, USA). PCNA (^#^GB11010), Histone H3 (^#^GB11102), F4/80 (^#^GB113373) and GAPDH (^#^GB11002) antibodies were purchased from Servicebio Biological Technology (Wuhan, China). Sirt3 (^#^10099‐1‐AP) and PGC‐1α (^#^66369‐1‐Ig) were obtained from Sanying Biotechnology (Wuhan, China). EED226 (^#^S8496) and cisplatin (^#^NSC119875) were purchased from Selleck Chemicals (Houston, TX, USA). Serum creatinine (^#^C011‐2‐1) and blood urea nitrogen (^#^C013‐2‐1) reagent kits were purchased from Nanjing Jiancheng Bioengineering Institute (Nanjing, China). All other chemicals were obtained from Beyotime (Shanghai, China).

### 2.2 Animals and treatment

Male C57BL/6J mice (20–23 g) were obtained from Shanghai SLAC Laboratory Animal Co. Ltd. (Shanghai, China). To establish a mouse model of cisplatin‐induced AKI, the animals were injected intraperitoneally with cisplatin (25 mg/kg) in saline. To study the effect of EED226 on AKI, EED226 (40 mg/kg) dissolved in corn oil and DMSO was administered intragastrically immediately after cisplatin injection, and then administered twice a day. The control group received the same amount of vehicle. Mice were euthanized at 48 h after cisplatin injection. Kidney samples were collected for histological examination, and the renal cortex was dissected and used for Western blot analyses. Serum was collected for measuring Scr and BUN. All experimental procedures were approved by the Institutional Animal Care and Use Committee of Shanghai East Hospital, Tongji University, China.

### 2.3 Renal function analysis

Scr and BUN levels were determined by Creatinine and BUN Kits, respectively, according to the manufacturer's instructions.

### 2.4 Assessment of tubular injury

A TUNEL staining kit was used to detect DNA strand breaks according to the instructions provided by Roche Molecular System (Branchburg, NJ). The number of TUNEL‐positive nuclei per field (*n* = 10) was evaluated.

### 2.5 Cell culture and treatment

Murine renal tubular epithelial cells (mRTECs) were cultured in DMEM/Nutrient F12 (DMEM/F12) containing 5% fetal bovine serum (FBS) and 0.5% penicillin and streptomycin in an atmosphere of 5% CO_2_–95% ambient air at 37°C. mRTECs at 60%–70% confluence were used for various treatments. To determine the effect of EED inhibition on cisplatin‐induced mRTEC cells injury, mRTEC cells were starved for 24 h with DMEM/F12 containing 0% FBS and then treated with EED‐226 (10 μM) or DMSO in the presence or absence of cisplatin (20 μg/ml) for 48 h.

### 2.6 Transfection

mRTECs were cultivated to 60%–70% confluence in culture medium containing no penicillin or streptomycin. EED, FoxO3a and p53 plasmid and empty vector (Genepharma Inc. Shanghai, China), or EED‐siRNA and control siRNA (Genepharma Inc. Shanghai, China) were transfected into the cells with Lipofectamine 3000 (#L3000001) (Invitrogen‐Thermo Fisher Scientific, Carlsbad, CA, USA), according to the manufacturer's protocol. At 6 h following transfection, the original antibiotic‐free medium was changed to DMEM/F12 containing 5% FBS and 0.5% penicillin and streptomycin, and cells were then exposed to cisplatin (20 μg/ml) for an additional 48 h before being harvested for the experiments.

### 2.7 Cell‐counting kit‐8 (CCK‐8) proliferation assay

Cell viability was determined using CCK‐8 assay (#GK10001) (Dojindo, Japan) according to the manufacturer's instructions. Cells were seeded in 96‐well plates and then transfected with siRNA and/or pretreated with EED226 for 30 min and then exposed to cisplatin (20 μg/ml). After treatment for 24 h, cells were exposed to CCK‐8 solution for 2 h. Absorbance at 490 nm (OD490) was detected by microplate reader.

### 2.8 qRT‐PCR

qRT‐PCR was carried out to detect the interleukin‐6 (IL‐6), Monocyte chemoattractant protein‐*1(*MCP‐1) and interleukin‐1β (IL‐1β) mRNA expression levels. Total RNA from tissues or cells was extracted using the TRIzol reagent (Invitrogen, CA, USA). Reverse transcription kit (TaKaRa, Tokyo, Japan) was used to synthesize cDNA. qRT‐PCR was performed with the qRT‐PCR kit (TaKaRa, Tokyo, Japan) by activating the DNA polymerase at 95°C for 5 min, followed by 40 cycles of two‐step PCR (at 95°C for 10 s and t 60°C for 30 s) and a final extension at 75°C for 10 min and held at 4°C. RNase‐free water was used as the templates of negative control. All primers were obtained from Sangon Biotech (Shanghai, China). The forward primer sequences of IL‐6 were F: 5′‐GCGGATCGGATGTTGTGAT‐3′; R: 5′‐GGACCCCAGACAATCGGTTG‐3′. Primer sequences for MCP‐1 were F: 5′‐AAAACACGGGACGAGAAACCC‐3′; R: 5′‐ACGGGAACCTTTATTAACCCCT‐3′. Primer sequences for IL‐1β were F: 5′‐ CAACTGTTCCTGAACTCAACT‐3′; R: 5′‐ATCTTTTGGGGTCCGTCAACT‐3′. Primer sequences for GAPDH were F: 5′‐ACTGCCATCTGCTCTCCACTTG‐3′; R: 5′‐CCTGGAAGATGGTGATGGGCTT‐3′. The formula 2^−ΔΔCT^ was applied to analyze the mRNA expression levels.

### 2.9 Western blot analysis

To prepare protein samples for western blotting, kidney tissue samples and cultured cells were homogenized in the presence of RIPA and a protease inhibitor cocktail. In brief, a total of 25 μg protein were separated by SDS‐P AGE gel electrophoresis and transferred to PVDF membrane in a tank. The membrane was blocked with 5% nonfat milk for 1 h at room temperature, and then incubated with specific primary antibodies at 4°C overnight. After being washed three times in TBS with Tween‐20, the membrane was incubated with a secondary antibody for 1 h at room temperature. After TBS rinsing, bound antibodies were visualized by fluorescence detection methods.

### 2.10 Histochemical and immunofluorescence staining

Tissues were fixed in 4.5% buffered formalin, dehydrated, and embedded in paraffin. Sections were stained with hematoxylin and eosin (HE). For immunofluorescent staining, primary antibodies against NGAL (1:200), SIRT3 (1:200), PGC‐1α (1:200), PCNA (1:200) and fluorescent‐conjugated secondary antibodies (1:300) were applied to the sections. Examination and scoring of sections from each kidney (*n* = 10 for each condition) were carried out in a blinded fashion. Tubular injury was scored on a scale from 0 to 3, where 0 = normal, 1 = injury <30%, 2 = injury 30%–60%, 3 = injury >60%.

### 2.11 Statistical analysis

Data obtained from this study were expressed as mean ± SEM. Statistical analyses were performed using one‐way anova, followed by a Newman–Keuls post‐test. The differences between two groups were compared by Student's t‐test using Prism 5.0 (GraphPad Software, San Diego, CA, USA). *p* < 0.05 was considered as statistically significant difference between mean values.

## 3 RESULTS

### 3.1 Blocking PRC2 activity with EED226 improves renal function and attenuates renal tubular cell injury in a murine model of cisplatin‐induced AKI

**FIGURE 1:**
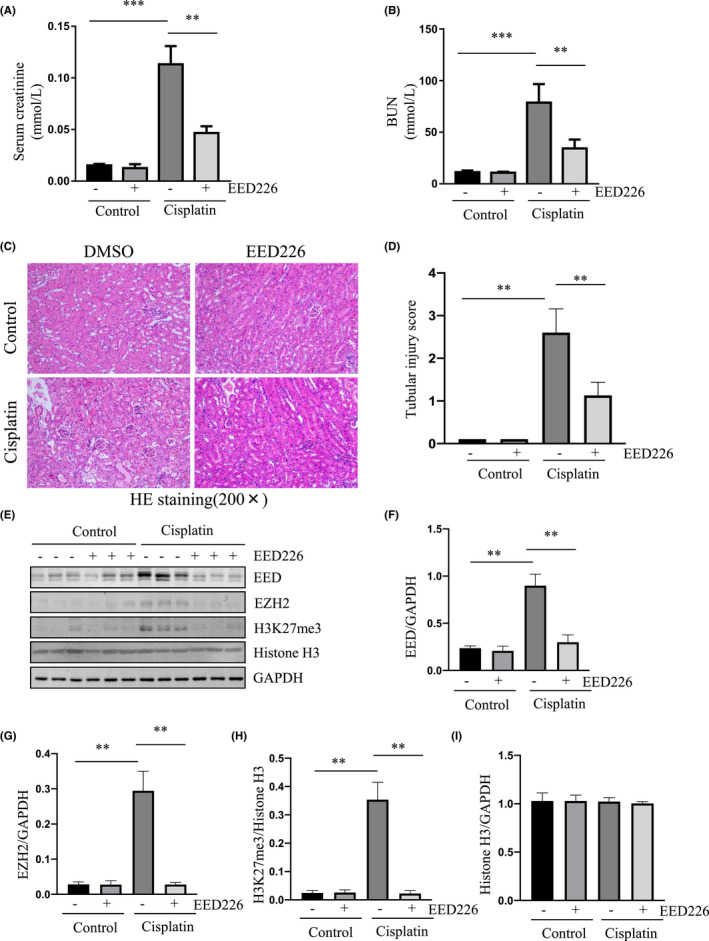
Blocking PRC2 activity with EED226 improves renal function and attenuates renal damage in a murine model of cisplatin‐induced AKI. Blood and kidneys were collected at 48 h after cisplatin injection with or without EED226. Serum creatinine (Scr) and blood urea nitrogen (BUN) were determined by the Assay kit as indicated in Materials and Methods (A, B). Photomicrographs illustrate HE stainning (C) and morphological changes were scored based on the scale described in Material and methods (D) The whole kidney lysates were subjected to immunoblot analysis with antibodies against EED, EZH2, and H3K27me3 (E). The levels of EED (F), EZH2 (G) and histone H3 (I) were quantified by densitometry and normalized with GAPDH. The levels of H3K27me3 were quantified by densitometry and normalized with Histone H3 (H). Values are the means ± SD (*n* = 6). Data are means ± SEM.*p < 0.05; **p < 0.01

EED protein is a core subunit of PRC2 that can promote the activity of PRC2 through its interaction with EZH2 and H3K27me3.^29^ Our recent studies demonstrated that inhibition of EZH2, the enzymatic unit of PRC2, protects against kidney damage in murine models of AKI induced by ischemia/reperfusion and folic acid^21^, ^22^; we thus assumed that inhibition of PRC2 catalytic activity by targeting EED would also protect against AKI. To test this hypothesis, we first examined the effect of EED226 on renal function of cisplatin‐induced AKI in mice by determining the changes in serum creatinine (Scr) and blood urea nitrogen (BUN) levels. Scr and BUN levels were elevated at 48 h in mice with cisplatin exposure compared to control groups whereas treatment with EED226 significantly reduced their levels (Figure 1A,B).

To determine the effect of PRC2 inhibition on kidney damage via renal tubular cell injury in mice injected with cisplatin, we examined the pathological changes and expression of NGAL, an early biomarker of AKI, in the kidneys. Kidney damage characterized by the presence of tubular dilatation, swelling, necrosis, and/or luminal congestion was observed in the kidneys of mice subjected to cisplatin, whereas administration of EED226 significantly reduced the renal tubular damage, as indicated by H&E staining (Figure 1C,D). Similarly, EED226 largely suppressed cisplatin‐induced expression of NGAL as shown by immunofluorescent staining (Figure S1) and confirmed by immunoblot analysis (Figure S1). Immunoblot analysis of the whole kidney lysates of those mice indicated that cisplatin‐induced renal dysfunction was coincident with increased expression of EZH2, EED, and H3K27me3; EED226 treatment dramatically reduced their expression as well (Figure 1E–H). Notably, expression levels of total histone H3 were not changed in the kidney of mice with cisplatin injection with/without EED226 treatment (Figure 1E,I). In addition, EED226 did not affect the basal level of EZH2, EED, and H3K27me3 (Figure 1E–H). Immunofluorescence staining confirmed the inhibitory effect of EED226 on the expression of EED in the kidneys of AKI mice (data not shown). These results suggest that inhibition of PRC2 catalytic activity by targeting EED can protect against cisplatin‐induced AKI in mice. Moreover, PRC2 positively regulates EZH2 and EED expression.

### 3.2 EED226 inhibition of PRC2 activity ameliorates renal tubular cell apoptosis in the kidney and cultured renal epithelial cells following cisplatin exposure

**FIGURE 2:**
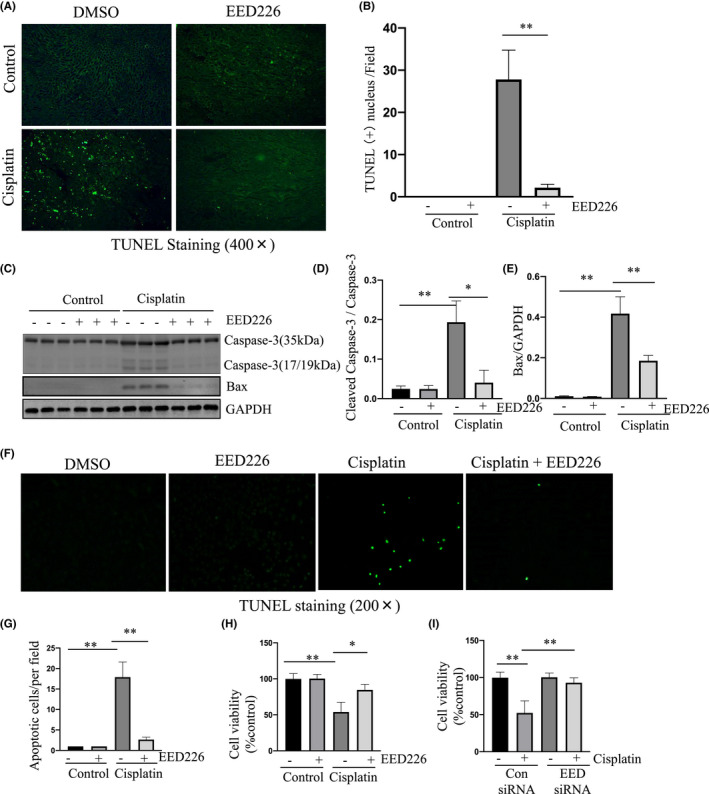
EED226 inhibits apoptosis of renal tubular cells in the murine kidney of cisplatin‐induced AKI. Photomicrographs illustrate TdT‐mediated dUTP nick‐end labeling (TUNEL) staining of the kidney tissues collected at 48 h after sham and cisplatin injection with or without EED226 in mice (A). Positive TUNEL staining cell nuclei were counted in 10 high‐power fields and expressed as means ± SD (B). The whole kidney lysates were subjected to immunoblot analysis with specific antibodies against Caspase‐3, Bax or GAPDH (C). Expression levels of cleaved Caspase‐3 (17/19 kDa) (D) were quantified by densitometry and normalized with total Caspase‐3 (35 kDa). Bax (E) were quantified by densitometry and normalized with GAPDH. mRTECs were pretreated with EED (10 μm) for 1 h (F–H) or transfected with control or EED siRNA for 24 h (I) and then exposed to cisplatin for 48 h. Cellular apoptosis was detected by TUNEL staining (F); TUNEL positive cells were calculated and expressed as the number of TUNEL positive cells /per field (G). Cell viability was determined by the CCK‐8 assay (H, I). Data are means ± SD.**p < 0.01, *n* = 6

Apoptotic cell death of renal epithelial cells is a prominent feature of AKI induced by nephrotoxic drugs including cisplatin.^10^ To understand the mechanism by which PRC2 activation contributes to renal damage, we examined the effect of EED226 on cisplatin‐induced apoptosis of renal tubular cells by TUNEL staining and immunoblot analysis of the expression of cleaved caspase‐3, a hallmark of apoptosis. In mice subjected to cisplatin, TUNEL positive renal tubular cells were observed in the kidney while EED226 treatment significantly reduced this population of cells. No apoptotic cells were observed in the kidney of Sham or EED alone treated kidneys. (Figure 2A,B). Immunoblot analysis confirmed that EED226 treatment effectively inhibited cisplatin‐induced cleavage of caspase‐3 and expression of Bax, a protein associated with mitochondrial dysfunction and AKI (Figure 2C–E). Moreover, we demonstrated that cisplatin treatment enhanced phosphorylation of MLKL (Mixed lineage kinase domain‐like) and RIPK3 (receptor‐interacting protein 3), two hallmarks of necroptosis, while EED226 treatment suppressed their phosphorylation (Figure S1).

**FIGURE 3:**
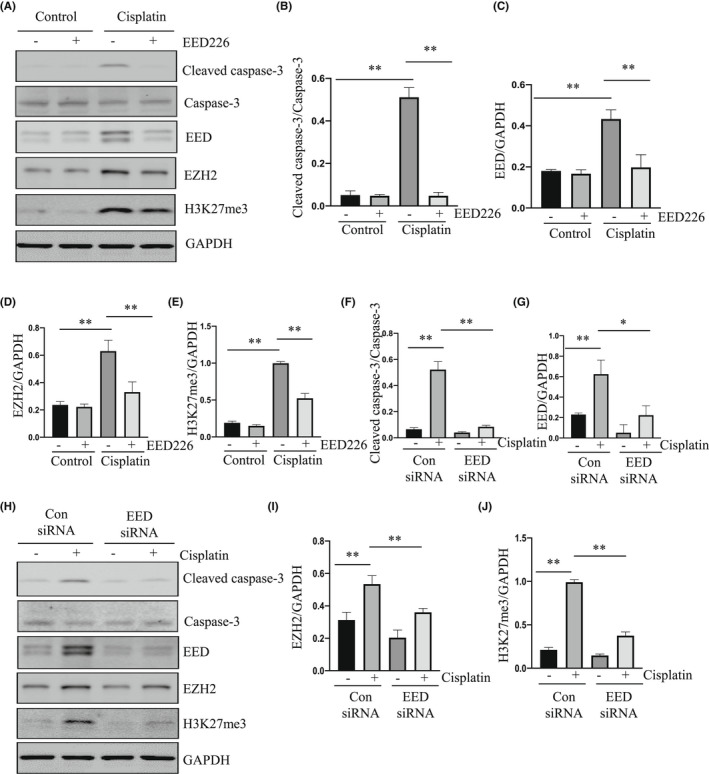
Treatment with EED226 or siRNA EED promotes cell survival and inhibits apoptosis in cultured mRTECs exposed to cisplatin. mRTECs were pretreated with EED226 (10 μM) for 1 h or transfected with control or EED siRNA for 24 h and then exposed to cisplatin for 48 h. Cell lysates were subjected to immunoblot analysis with specific antibodies against cleaved Caspase‐3, caspase‐3, EED, EZH2, H3K27me3 (A, H). Expression of cleaved caspase‐3 (B, F) were quantified by densitometry and normalized with total caspase‐3. EED (C, G), EZH2 (D, I), H3K27me3 (E, J) were quantified by densitometry and normalized with GAPDH. Data are means ± SD.*p < 0.05, **p < 0.01, *n* = 6

To validate the effect of EED inhibition on the apoptosis of renal tubular epithelial cells, we determined the effect of EED226 or siRNA specific to EED on cell viability and/or apoptosis of mRTECs following cisplatin exposure in culture. Consistent with what we had observed in the kidney of mice, treatment with EED226 also significantly inhibited apoptosis (Figure 2F,G), and both EED226 and EED siRNA promoted cell survival (Figure 2H,I) in cells exposed to cisplatin. Moreover, EED226 and EED siRNA were effective in suppressing cisplatin‐induced cleavage of caspase‐3, which was coincident with reduced expression of EED, EZH2 and H3K27me3 (Figure 3A–J).

To further validate the role of EED in mediating apoptosis of renal tubular epithelial cells, we conducted a rescue experiment by overexpressing EED in cultured mRTECs followed by cisplatin exposure in the presence or absence of EED226. As shown in Figure S1, overexpression of exogenous EED led to increased expression of H3K27me3 and cleaved caspase‐3 and elevated apoptosis, and diminished the inhibitory effect of EED‐226 on all these events in cells exposed to cisplatin.

Taken together, our results illustrated that PRC2 activity is essential for apoptosis and necroptosis and activation of apoptotic and necroptotic signaling molecules in cisplatin‐induced AKI.

### 3.3 Inhibition of PRC2 activity by EED226 or EED siRNA reduces phosphorylation of p53 and FoxO3a in the kidney and cultured renal epithelial cells following cisplatin exposure

**FIGURE 4:**
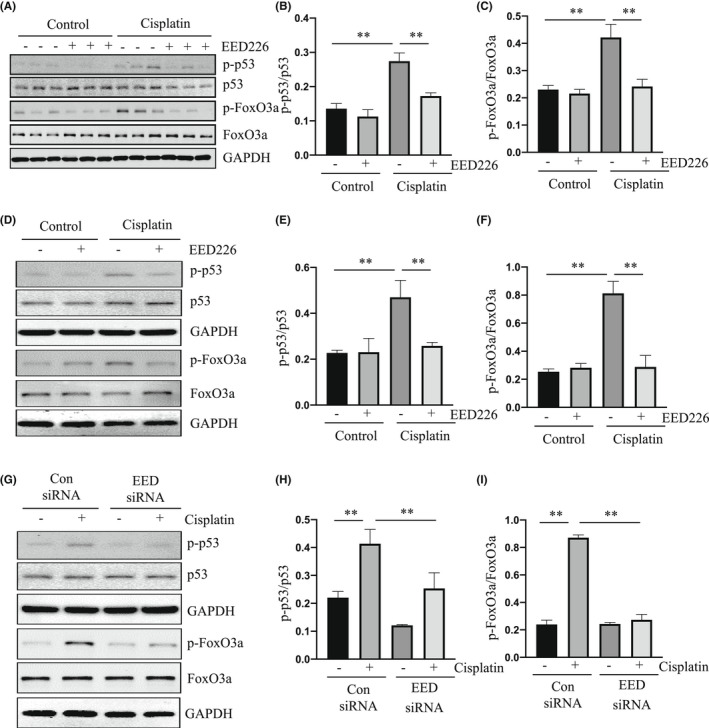
EED226 or EED siRNA reduces phosphorylation of p53 and FoxO31 in the kidney and cultured mRTECs following cisplatin exposure. Kidneys were collected at 48 h after cisplatin injection with or without EED226. The whole kidney lysates were subjected to immunoblot analysis with specific antibodies against p‐p53(Ser15), p53, p‐FoxO3a (Ser 253), FoxO3a, or GAPDH (A). Expression of p‐p53 (B) and p‐FoxO3a (C) were quantified by densitometry and normalized with p53 and FoxO3a, respectively. mRTECs were pretreated with EED226 (10 μM) for 1 h (D) or transfected with control or EED siRNA for 24 h (G) and then exposed to cisplatin for 48 h. Cell lysates were subjected to immunoblot analysis with specific antibodies against p‐p53, p53, p‐FoxO3a, FoxO3a, or GAPDH. Expression of p‐p53 (E, H) and p‐FoxO3a (F, I) were quantified by densitometry and normalized with p53 and FoxO3a, respectively. Data are means ± SD. **p < 0.01, *n* = 6

p53 is a critical mediator in cisplatin‐induced AKI,^10^ and Forkhead box O3a (FoxO3a) acts downstream of p53 to contribute to mitochondrial dysfunction.^30^, ^31^ To elucidate whether PRC2 would mediate apoptosis through a mechanism involved in the activation of the p53 pathway, we examined the effect of EED226 or EED siRNA on the phosphorylation and expression of p53 and FoxO3a in the kidney and cultured renal epithelial cells following cisplatin exposure. Figure 4A–C demonstrates that cisplatin‐induced AKI was accompanied by increased expression levels of p‐p53 at serine 15 and p‐FoxO3a at serine 253 while total levels of p53 and FoxO3a remained unchanged. EED226 treatment significantly inhibited p53 and FoxO3a phosphorylation without affecting expression of total p53 and FoxO3a. In line with those observations, treatment with EED226 or EED siRNA also inhibited cisplatin‐induced phosphorylation of p53 and FoxO3a in cultured mRTECs (Figure 4D–I).

To further understand the role of p53 or FoxO3a in renal tubular epithelial cell apoptosis and their relation to EED, we examined the effect of p53 or FoxO3a overexpression on the apoptosis of this cell type in culture exposed to cisplatin in the presence and absence of EED226. Our data demonstrated that overexpression of either p53 (Figure S1) or FoxO3a (Figure S1) enhanced cisplatin‐induced cleavage of caspase‐3 and reduced the inhibitory effect of EED226 on this event. Moreover, overexpression of p53 and FoxO3a increased p53 and FoxO3a phosphorylation, respectively, and decreased the inhibitory effect of EED226 on phosphorylation of each of them (Figure S1).

Collectively, these data suggest that PRC2 may contribute to mitochondrial dysfunction and apoptosis through a mechanism involved in the activation of the p53/FoxO3a signaling pathway.

### 3.4 PRC2 activity is required for retaining the expression of Sirt3 and PGC‐1α in the kidney of mice and cultured renal epithelial cells following cisplatin exposure

**FIGURE 5:**
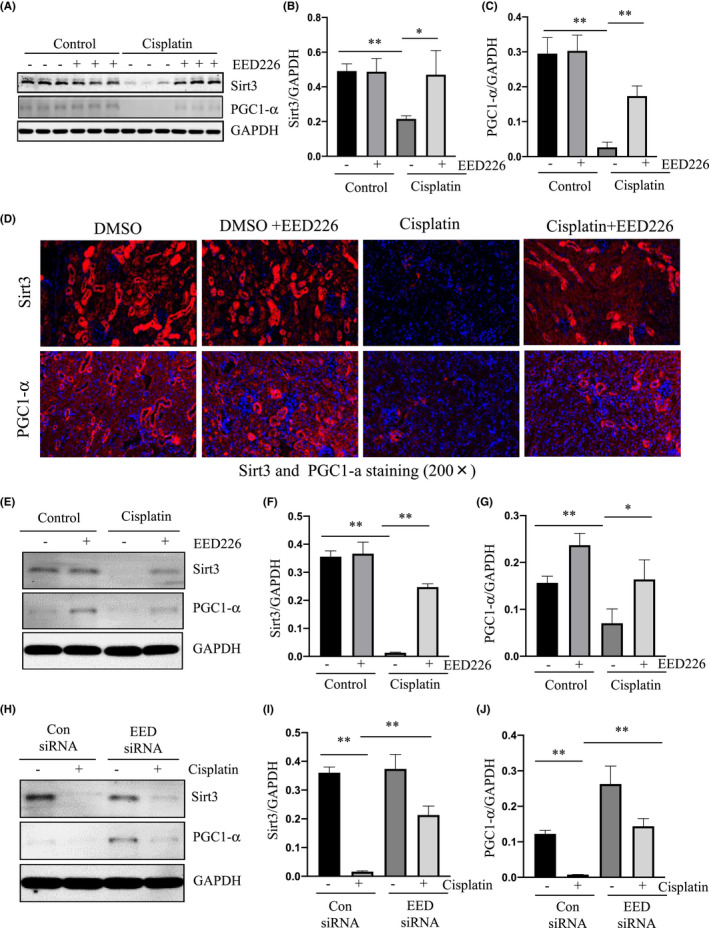
Treatment with EED226 or EED siRNA preserves expression of Sirt3 and PGC‐1α in the kidney and cultured mRTECs following cisplatin exposure. Kidneys were collected at 48 h after cisplatin injection with or without EED226. The whole kidney lysates were subjected to immunoblot analysis with specific antibodies against Sirt3 and PGC‐1a or GAPDH (A). Expression of Sirt3 (B) and PGC‐1α (C) were quantified by densitometry and normalized with GAPDH. Photomicrographs illustrate fluorescent staining of SIRT3 and PGC‐1α in mouse kidney sections (D). mTECs were pretreated with EED226 (10 μM) for 1 h (E) or transfected with control or EED siRNA for 24 h (H) and then exposed to cisplatin for 48 h. Cell lysates were subjected to immunoblot analysis with specific antibodies against Sirt3, PGC‐1α or GAPDH. Expression levels of Sirt3 (F, I) or PGC‐1α (G, J) were quantified by densitometry and normalized with GAPDH. Data are means ± SD.*p < 0.05, **p < 0.01, *n* = 6

Mitochondrial damage is closely connected to apoptosis, which is regulated by damage and protective signaling molecules.^13^ It has been reported that both mitochondrial sirtuin (SIRT3) and peroxisome proliferator‐activated receptor‐gamma coactivator‐1α (PGC‐1α) play a role in maintaining the stability of mitochondrial structure and function, thereby offering a renoprotective effect in the injured kidney.^32^ In addition, SIRT3 activation can increase the expression of PGC‐1α in AKI induced by cisplatin and reduce mitochondrial fragmentation and renal function damage.^16^ On this basis, we proceeded to examine the effect of EED226 on the expression of SIRT3 and PGC‐1α in the kidneys of mice with cisplatin‐induced AKI. In the kidneys of mice with cisplatin‐induced AKI, the expression of SIRT3 and PGC‐1α was significantly reduced relative to control kidneys, while EED226 treatment led to a significant preservation of SIRT3 in the injured kidney. Similarly, EED226 treatment also partially restored expression levels of PGC‐1α in the kidney with cisplatin exposure (Figure 5A–D). In cultured mRTECs, treatment with either EED226 or EED siRNA was also effective in suppressing cisplatin‐induced downregulation of SIRT3 and PGC‐1α as shown by immunoblot analysis (Figure 5E–J). Based on these results, we suggest that EED226 elicited renoprotection may also be associated preservation of SIRT3 and PGC‐1α in renal epithelial cells following cisplatin exposure.

### 3.5 EED226 treatment promotes proliferation of renal tubular epithelial cells in a murine model of cisplatin‐induced AKI

**FIGURE 6:**
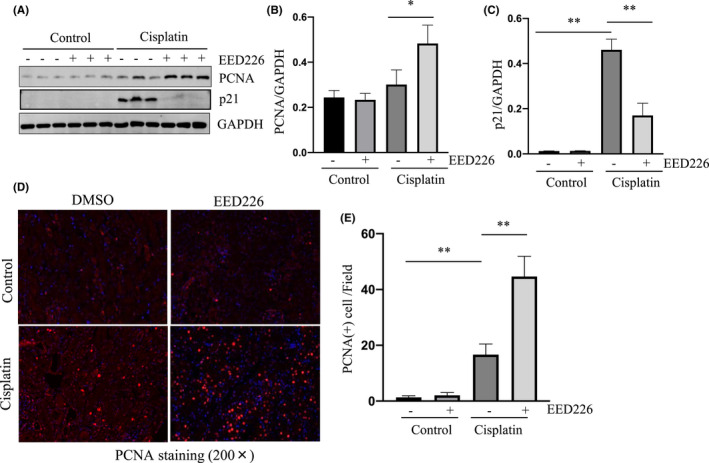
EED226 promotes the proliferation of renal tubular epithelial cells in a mouse model of AKI induced by cisplatin. Kidneys were collected at 48 h after cisplatin injection with or without EED226. The whole kidney lysates were subjected to immunoblot analysis with specific antibodies against PCNA, p21 and GAPDH (A). Expression levels of PCNA (B) and p21(C) were quantified by densitometry and normalized to GAPDH. Photomicrographs illustrate fluorescent staining of PCNA in mouse kidney sections (D). The number of PCNA positive staining cells in mouse kidney sections was calculated (E). Data are means ± SD.*p < 0.05, **p < 0.01, *n* = 6

The proliferation of renal tubular epithelial cells is a necessary step for renal regeneration following AKI. Increased expression of proliferating cell nuclear antigen (PCNA) is involved in cell cycle progression whereas the inhibitor of cyclin‐dependent kinase p21 is a blocker of the G1→S phase of cells that is associated with inhibition of cell proliferation.^33^, ^34^, ^35^ As such, we further examined the effect of EED inhibition on the expression of PCNA and p21, in the kidneys of mice with/without cisplatin exposure. In the cisplatin‐injured kidney, expression levels of PCNA were slightly increased whereas p21 expression levels were significantly increased compared with those in control kidneys. Interestingly, administration of EED226 significantly increased the expression of PCNA, but largely reduced p21 expression in the cisplatin injured kidneys (Figure 6A–C). The promoting effect of EED226 on the proliferation of renal cellular cells was also demonstrated by immunofluorescence staining (Figure 6D,E) as the number of PCNA (+) cells in the kidneys of cisplatin‐injured mice treated with EED226 was greatly increased compared to kidneys that weren't treated with EED226. The different effects of EED226 on the expression of these molecules suggest that EED226 treatment can promote the proliferation of renal tubular epithelial cells after AKI.

### 3.6 EED226 inhibits phosphorylation of STAT3 and NF‐κB and reduces infiltration of inflammatory cells into kidneys after cisplatin exposure

**FIGURE 7:**
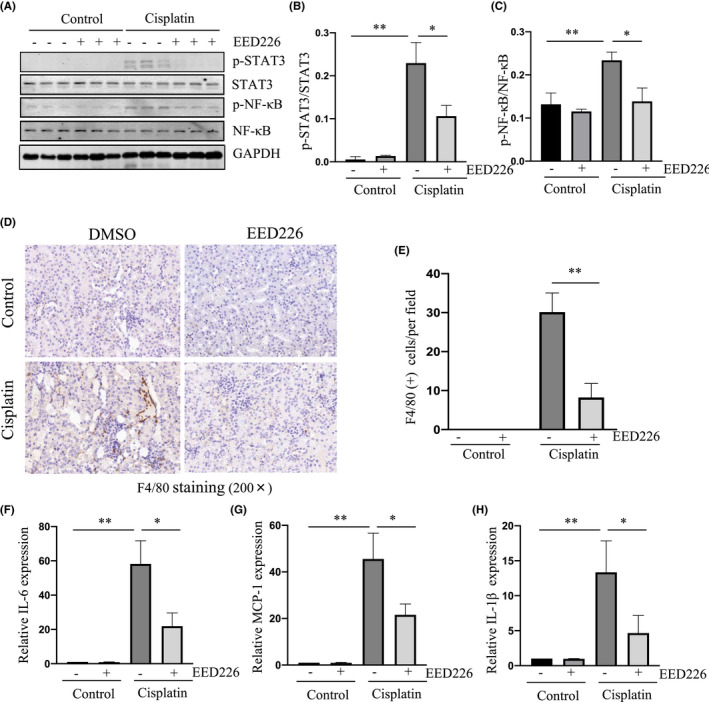
EED226 inhibits phosphorylation of STAT3 and NF‐κB and reduces infiltration of macrophages and release of inflammatory factors in cisplatin‐induced AKI mouse kidneys. Kidneys were collected at 48 h after cisplatin injection with or without EED226. The whole kidney lysates were subjected to immunoblot analysis with specific antibodies against p‐STAT3 (Tyr 705), STAT3, p‐NF‐kB (Ser 536), NF‐kB or GAPDH (A). Expression of p‐STAT3 (B) and p‐NF‐kB (C) were quantified by densitometry and normalized with STAT3 and NF‐kB, respectively. Photomicrographs illustrate immunochemical staining of F4/80 in the mouse kidney section (D). The number of positive cells stained with F4/80 was calculated in a mouse kidney section (E). qRT‐PCR assay of the mRNA expression levels of IL‐6 (F), IL‐1β (G) and MCP‐1 (H) in the kidney. Data are means ± SD. *p < 0.05, **p < 0.01, *n* = 6

Inflammation plays an important role in the occurrence and development of AKI. Two transcriptional factors, STAT3 and NF‐κB, have been demonstrated to be involved in the inflammatory response in a murine model of cisplatin‐induced AKI.^36^, ^37^, ^38^, ^39^ We hypothesize that EED may contribute to inflammatory response through regulation of STAT3 and NF‐κB phosphorylation in the injured kidney after cisplatin administration. As expected, cisplatin induced phosphorylation of both STAT3 (Tyr 705) and NF‐κB (Ser 536) whereas EED226 treatment significantly inhibited their phosphorylation (Figure 7A–C). Immunohistochemical examination also showed an increase in F4/80‐labeled macrophages in the acutely injured kidney tissue of mice exposed to cisplatin compared to those in the control group; EED226 treatment significantly reduced this population of macrophages in the kidney of AKI mice (Figure 7D,E). In line with this observation, EED226 was also effective in suppressing renal expression of multiple proinflammatory factors, including IL‐6, MCP‐1, and IL‐1β in cisplatin exposed animals (Figure 7F–H). Therefore, EED mediated PRC2 activation is involved in the regulation of cisplatin‐induced renal inflammation in AKI mice, and its mechanism may be related to the activation of STAT3 and NF‐κB signaling pathways.

## 4 DISCUSSION

Cisplatin is effective in treating various solid tumors, but its application is limited due to serious side effects, including nephrotoxicity.^10^ Recently, we found that EZH2 plays a critical role in the pathogenesis of AKI.^21^, ^22^ Given that EZH2 is the enzymatic unit of PRC2 and the catalytic activity of PRC2 is dependent on the interaction between EZH2 and EED, we here investigated whether disruption of PRC2 by inhibiting EED would affect renal function and pathological changes in a murine model of cisplatin‐induced AKI. Our results demonstrated that inhibition of the PRC2 activity by targeting EED with EED226 can lessen renal damage and improve renal function by promoting the survival of renal tubular epithelial cells. Moreover, specific silencing of EED by siRNA inhibited apoptosis of cultured mRTECs. We have thus provided strong evidence that inhibition of PRC2 activity by targeting EED would be a novel strategy to treat AKI.

The function of EED within PRC2 is mediated by a complex network of protein–protein interactions.^40^ Allosteric activation of PRC2 by binding of EED with methylated proteins such as H3K27 is essential for its catalytic activity.^41^ It has been reported that EED226 can directly bind to the H3K27me3 binding pocket of EED and interrupt the interaction of EZH2 with H3K27me3.^42^ Our results demonstrated that treatment with EED226 in vivo and in vitro largely reduced the expression levels of H3K27me3, and specifically silencing EED with siRNA in cultured mRTECs also decreased expression of H3K27me3, supporting the importance of EED in the enzymatic activity of PRC2. In addition, we found that treatment with EED226 and EED siRNA not only reduced the expression of H3K27me3, but also expression of EZH2 and EED. These data, together with our previous observations that inhibition of EZH2 with its inhibitors (DZNep or GSK126) or siRNA reduces the expression of EZH2, suggest that PRC2 activation triggers a feedback mechanism that positively regulates its own expression and activation. Currently, such a feedback machinery remains unclear. Since EZH2 expression is known to be regulated by multiple mechanisms, including ubiquitination, miRNA, and long‐chain non‐coding miRNA,^43^ further investigations are needed to identify the factors and mechanisms by which PRC2 activation regulates expression of its subunits, including EZH2 and EED.

Although multiple mechanisms contribute to the pathogenesis of cisplatin nephrotoxicity, accumulating evidence has suggested that mitochondrial and DNA damage‐induced apoptosis of renal tubular cells play a critical role.^12^ As Bax binding to the outer mitochondrial membrane induces the release of cytochrome C and other pro‐apoptotic factors that promote the activation of caspases,^44^ we initially examined the effect of EED inhibition on the expression of Bax and activation of caspase‐3, a key mediator of apoptosis, and found that treatment with either EED226 or EED siRNA reduces cisplatin‐induced Bax upregulation and caspase‐3 cleavage. This suggests that PRC2 may contribute to renal cell apoptosis through activation of the intrinsic pathway involved in mitochondrial damage. In addition, pharmacological and genetic inhibition of EED suppressed phosphorylation of p53, one of the key effectors mediating DNA damage response (DDR), in the kidney and cultured mRTECs exposed to cisplatin. This further suggests that PRC2 may be able to promote renal cisplatin nephrotoxicity through DDR initiated activation of p53. In this context, the role of p53 in cisplatin‐AKI has been well established by various pharmacological and genetic inhibitory approaches.^45^ P53 can induce apoptosis through regulation of several downstream effectors, including FoxO3a via transcription dependent and independent mechanisms.^31^, ^46^, ^47^In this study, we observed that treatment with EED226 and EED siRNA blocked phosphorylation of FOXO3a in the kidney and cultured mRTECs following cisplatin exposure, while overexpression of either p53 or FOXO3a reduced the inhibitory effect of EED226 on caspase‐3 cleavage. Thus, we suggest that cisplatin injury may induce renal epithelial cell death and AKI at least in part through activation of the PRC2‐p53‐FOXO3a signaling pathway.

PRC2‐EZH2 may also promote AKI by downregulation of renal protective proteins. In addition to the above‐mentioned proapoptotic protein molecules, mitochondria express renal protective proteins such as SIRT3 and PGC‐1α.^16^, ^48^ SIRT3 is an NAD‐dependent protein deacetylase that regulates energy metabolism through deacetylation and activation of mitochondrial acetyl‐CoA synthase^49^, ^50^; PGC‐1α is an important transcriptional co‐activator that is involved in the regulation of energy metabolism through promoting mitochondrial biogenesis.^51^ Our results demonstrate that EED226 treatment partially prevents cisplatin‐induced downregulation of both SIRT3 and PGC‐1α in the kidney and in cultured mRTECs, suggesting that PRC2 may mediate apoptosis and AKI through a negative regulation of these two transcriptional factors. In line with our observations, a previous study demonstrated that loss‐of‐function mutation in EED can increase the resistance of T cell mitochondria to chemical damage.^52^ As such, PRC2 inhibition may suppress the mitochondrial damage and apoptosis of renal tubular cells by reducing not only activation or expression of mitochondrial damage‐promoting molecules, but also by restoring and/or promoting the expression of some renal protective proteins such as SIRT3 and PGC‐1α.

Induction of AKI by various insults is accompanied by severe inflammatory response. During AKI, tubular cell death leads to the release of inflammatory factors and inflammatory cell infiltration to the kidney.^53^ Some proinflammatory factors such as TNF‐α not only promote cell apoptosis and enhance kidney damage, but also activate inflammatory signaling pathways such as STAT3 and NF‐κB to trigger the inflammatory response.^36^, ^37^, ^38^, ^39^, ^54^ In this study, we found that inhibition of PRC2 activity by EED226 significantly reduced cisplatin‐induced STAT3 and NF‐κB phosphorylation and macrophage infiltration. Furthermore, EED226 effectively inhibited the expression of several inflammatory factors including IL‐6, MCP‐1, and IL‐1β. Therefore, reduction of inflammatory responses would be another mechanism of EED226‐elicited renoprotection following cisplatin exposure.

Unlike the heart and brain, the kidney has a great ability to regenerate after injury.^55^ At the time when renal epithelial cell death occurs after AKI, the tubular repair and regenerative response is also triggered.^56^ In the current study, we observed that cisplatin‐induced AKI was accompanied with a slight increase in the expression of the cell proliferation marker PCNA, and EED226 treatment further increased PCNA expression and inhibited the expression of cell cycle inhibitor protein p21. Therefore, EED226‐mediated improvement in renal functional may also be related to its actions promoting regeneration and repair of renal tubular cells. Further studies are needed to elucidate the mechanism by which inhibition of PRC2‐EZH2 promotes such beneficial effects in the injured kidney.

Numerous studies have shown that EZH2 gene mutations or dysfunction of PRC2 is associated with development of a variety of human tumors.^40^ Inhibiting the activity of PRC2 has become an important approach for the treatment of cancer. The catalytic subunit of PRC2 is EZH2, which regulates gene expression by catalyzing H3K27 trimethylation (H3K27me3).^26^ Therefore, most of initial drug discovery was focused on developing small molecules that target the SET domain of EZH2 activity that are for trimethylation of H3K27 or use competitive inhibitors of S‐adenosylmethionine (SAM), the methyl donor essential for the HMT activity. In the past two decades, a variety of EZH2 small molecule inhibitors have been synthesized and tested in different animal models of tumors.^40^ Among them, tasemetostat has been recently approved by the FDA for the treatment of epithelioid sarcoma.^40^ However, since SAM not only provides the methyl donor to the substrates of EZH2, but also to the substrates of other HMTs, lacking specificity,^40^ and secondary mutations of EZH2 could also cause drug resistance, targeting the allosteric site of EED may provide a specific and alternative approach to inhibit PRC2 activity. Based on the current research and our previous investigations showing that effective inhibition of PRC2 catalytic activity can be achieved by blocking either EED or EZH2 in different models of AKI,^21^, ^22^ we suggest that pharmacologically targeting PRC2 may be a novel approach to treat AKI induced by various insults.

In summary, our results have demonstrated that PRC2 is a critical regulator of renal injury in cisplatin‐induced nephropathy and that inhibition of its activity by pharmacological targeting EED can significantly improve renal function and ameliorate renal tubular cell apoptosis and other pathological responses like macrophage infiltration. As nephrotoxicity is a major adverse effect limiting the use of cisplatin and its derivatives in cancer therapy, and PRC2 contributes to both nephrotoxicity and tumorgenesis, it is reasonable to speculate that inhibition of PRC2 activity in tumor patients treated with cisplatin would not only alleviate its nephrotoxicity, but also enhances its anti‐tumor effects, achieving the goal of “hitting two birds with one stone”. Therefore, allosterically targeting PRC2 activity by inhibiting EED action may offer a new and effective approach for renoprotection during cisplatin‐based cancer therapy. Further investigations by combined application of PRC2 inhibitors and cisplatin in animal models of tumor will provide evidence of this.

## AUTHOR CONTRIBUTIONS

**Chao Yu:** Conceptualization (equal); data curation (equal); formal analysis (equal); investigation (equal); methodology (equal); validation (equal); visualization (equal). **Tingting Li:** Data curation (equal); investigation (equal); methodology (equal); writing – original draft (equal). **Jialu Li:** Investigation (equal); methodology (equal); validation (supporting). **Binbin Cui:** Investigation (equal); validation (supporting). **Na Liu:** Conceptualization (equal); methodology (equal); project administration (equal); validation (supporting). **George Bayliss:** Validation (equal); writing – review and editing (equal).

## CONFLICT OF INTERTEST

The authors declare that they have no conflict of interest.

## Supporting information


Figure S1

## Data Availability

The data that support the findings of this study are available from the corresponding author upon reasonable request.
