# Supplementary material for: Inhibition of polycomb repressive complex 2 by targeting EED protects against cisplatin‐induced acute kidney injury
Source: J Cell Mol Med. 2022 Jun 23;26(14):4061–75. doi: 10.1111/jcmm.17447 (PMC9279598; doi:10.1111/jcmm.17447)
Supplement: Supplementary file 1 — Figure S1 [file JCMM-26-4061-s001.docx]

**
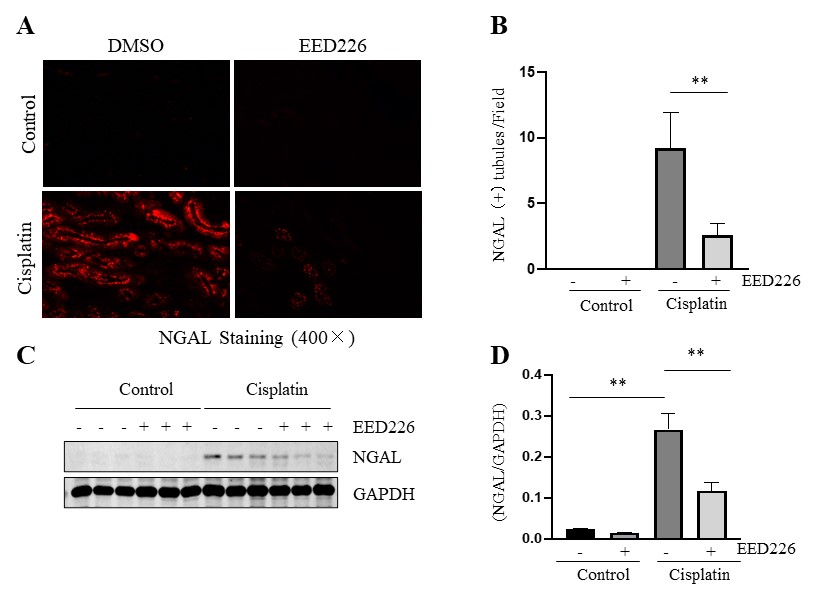
**

**Supplemental Figure 1. Inhibition of PRC2 by EED226 reduces renal tubule injury in a murine model of cisplatin-induced AKI.** Kidneys were collected at 48 hours after cisplatin injection with or without EED226. Photomicrographs illustrating immunofluorescent staining of NGAL in mouse kidney sections (A). Tubules with positive NGAL staining were counted in 10 high-power fields and expressed as means ± SD (B). The whole kidney tissue lysates were subjected to immunoblot analysis with specific antibodies against NGAL and GAPDH (C). Expression levels of NGAL were quantified by densitometry and normalized with GAPDH (D). Data are means ± SD**P < 0.01, n=6.


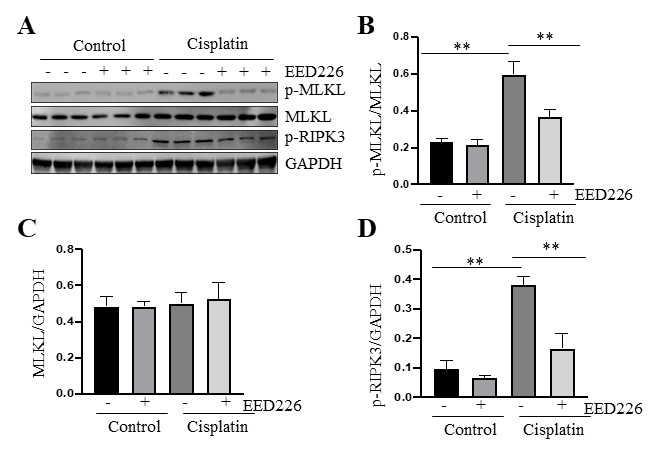


**Supplemental Figure 2．EED226 inhibits phosphorylation of MLKL and RIPK3, two necroptotic markers, in the kidney of mice following cisplatin treatment.** Kidneys were collected at 48 hours after cisplatin injection with or without EED226. The whole kidney lysates were subjected to immunoblot analysis with specific antibodies against p-MLKL (Ser345), MLKL, p-RIPK3 (Thr231/Ser232) or GAPDH (A). Expression of p-MLKL (B), MIKL (C) and p-RIPK3 (D) were quantified by densitometry and normalized with MLKL and GAPDH, respectively. Data are means ± SD. **P<0.01, n=6.


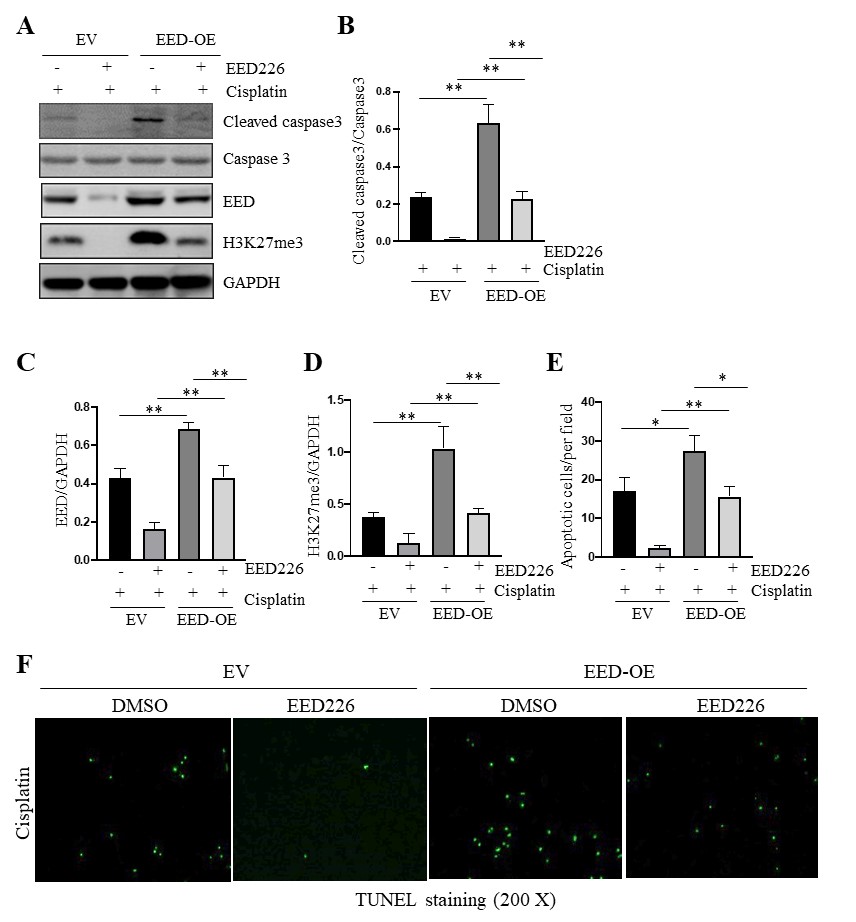
**Supplemental Figure 3. Overexpression of EED (EED-OE) enhances caspase 3 cleavage and diminishes the inhibitory effect of EED226 on the cleavage of Caspase3 in cultured mRTECs following cisplatin exposure.** mRTECs were transfected with control or EED-expression vectors as indicated in Materials and Methods and then exposed to cisplatin for 48 hours in the absence or presence of EED226 (10 μM). Cell lysates were subjected to immunoblot analysis with specific antibodies against Cleaved caspase 3, Caspase 3, EED, H3K27me3 or GAPDH (A). Expression levels of Cleaved caspase 3 (B), EED (C) H3K27me3 (E) were quantified by densitometry and normalized with Caspase 3 or GAPDH, respectively. TUNEL (+) cells were calculated and expressed as the number of TUNEL apoptotic cells /per field (E). Photographs showing TUNEL staining (F). Data are means ± SD.*P<0.05, **P<0.01, n=6. EV: Empty vector.

**
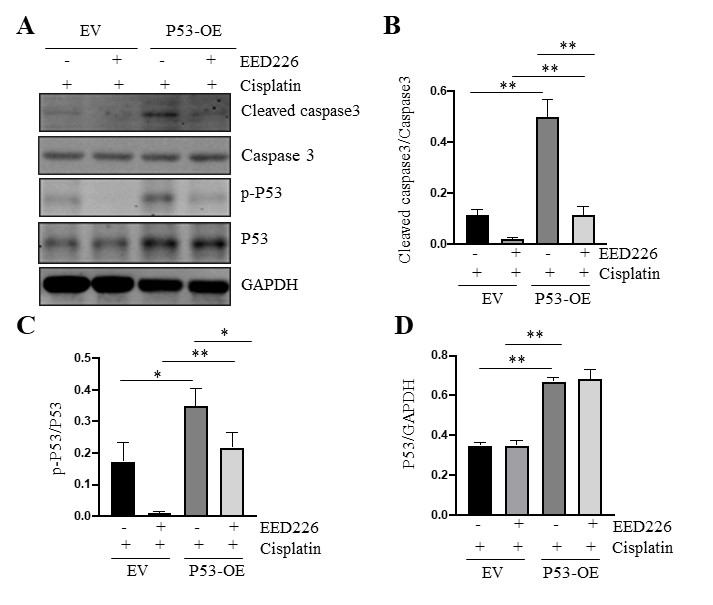
**

**Supplemental Figure 4. Overexpression of p53 (p53-OE) enhances caspase3 cleavage and partially diminishes the inhibitory effect of EED226 on the cleavage of caspase3 in cultured mRTECs following cisplatin exposure.** mRTECs were transfected with control or P53-expression vectors as indicated in Materials and Methods and then exposed to cisplatin for 48 hours in the absence or presence of EED226 (10 μM). Cell lysates were subjected to immunoblot analysis with specific antibodies against Cleaved caspase3, Caspase3, p-p53, p53 or GAPDH (A). Expression levels of Cleaved caspase3 (B), p-p53 (C), p53 (D) were quantified by densitometry and normalized with caspase3, p53 or GAPDH, respectively. Data are means ± SD.*P<0.05, **P<0.01, n=6. EV: Empty vector.

**
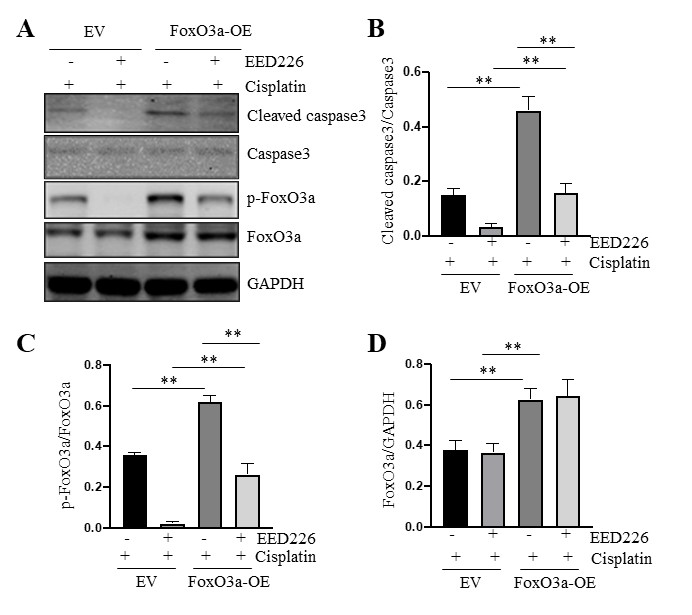
**

**Supplemental Figure 5. Overexpression of FoxO3a (FoxO3a-OE) enhances caspase-3 cleavage and diminishes the inhibitory effect of EED226 on the cleavage of caspase3 in cultured mRTECs following cisplatin exposure.** mRTECs were transfected with control or FoxO3a expression vectors for 24 hours and then exposed to cisplatin for 48 hours in the absence or presence of EED226 (10 μM). Cell lysates were subjected to immunoblot analysis with specific antibodies against Cleaved caspase3, Caspase3, p-FoxO3a, FoxO3a or GAPDH (A). Expression levels of Cleaved caspase 3 (B), p-FoxO3a (C), FoxO3a (E) were quantified by densitometry and normalized with caspase 3, FoxedO3a or GAPDH, respectively. Data are means ± SD. **P<0.01. n=6. EV: Empty vector.
